# Evaluating a model for the capacity building of midwifery educators in Bangladesh through a blended, web-based master’s programme

**DOI:** 10.1080/16549716.2019.1652022

**Published:** 2019-08-14

**Authors:** Kerstin Erlandsson, Ulrika Byrskog, Fatumo Osman, Christina Pedersen, Mathias Hatakka, Marie Klingberg-Allvin

**Affiliations:** a School of Education, Health and Social Studies, Dalarna University, Falun, Sweden; b School of Technology and Business Studies, Dalarna University, Falun, Sweden

**Keywords:** Midwifery education, capacity building, web-based education, Bangladesh, South Asia

## Abstract

**Background**: While setting international standards for midwifery education has attracted considerable global attention, the education and training of midwifery educators has been relatively neglected, particularly in low-resource settings where capacity building is crucial. **Objective**: The aim of this study was to describe the expectations of midwifery educators in Bangladesh who took part in a blended web-based master’s programme in SRHR and the extent to which these were realized after 12 months of part-time study.

**Methods**: Both quantitative and qualitative methods have been used to collect data. A structured baseline questionnaire was distributed to all participants at the start of the first course (n = 30) and a second endpoint questionnaire was distributed after they (n = 29) had completed the core courses one year later. At the start of the first course, five focus group discussions (FGD) were held with the midwifery educators. Descriptive statistics and content analysis were used for the analyses.

**Results**: Midwifery educators who took part in the study identified expectations that can be grouped into three distinct areas. They hoped to become more familiar with technology, anticipated they would learn pedagogical and other skills that would enable them to better support their students’ learning and thought they might acquire skills to empower their students as human beings. Participants reported they realized these ambitions, attributing the master’s programme with helping them take responsibility for their own teaching and learning, showing them how to enhance their students’ learning and how to foster reflective and critical thinking among them.

**Conclusions**: Midwifery educators have taken part in a creative learning environment which has developed their engagement in teaching and learning. They have done this using a blended learning model which combines online learning with face-to-face contact. This model can be scaled up in low resource and remote settings.

## Background

Maternal and child mortality and morbidity remain major public health concerns in most low-resource settings. Both are dependent upon the presence of qualified midwifery care but there is often a shortage of midwives who are adequately trained and educated according to international standards [1]. The midwifery workforce plays a critical role in meeting women’s sexual, reproductive, maternal and newborn healthcare needs [2,3]. In order to ensure midwives are adequately trained [4–6], high quality midwifery education is essential. While the International Confederation of Midwives (ICM) and the World Health Organization (WHO) have set up international standards for midwifery education, researchers havent’t paid much attention to this, but global policymakers have. Thus, building the capacity of midwifery educators international standards for midwifery education have been laid out by the WHO in a set of ‘core competencies’ and by the ICM standards for education [7,8]. The UNFPA’s newly-launched midwifery strategy, part of its wider strategic plan, contains further guidelines on building the capacity of midwifery educators [9].

Bangladesh is a lower middle-income country with approximately 3.1 million live births per year [10]. Most people live in rural areas where the dispersed nature of the population and a general shortage of skilled midwives contributes to a relatively low figure of 50 percent skilled attendance at births and a high maternal mortality ratio (MMR) of around 196 maternal deaths for every 100,000 live births [11]. The government of Bangladesh has sought to address the high MMR in particular by introducing midwifery as a separate health profession dedicated to reproductive, maternal and newborn healthcare. In 2010 the government of Bangladesh called for the education and deployment of 3,000 midwives. Prior to this there had been no educational programme that prepared professional midwives according to international standards. Instead, midwifery was taught as small part of a wider nursing degree. Nurses could obtain a dual degree in nursing and midwifery and work as nurse-midwives. Starting in 2010 specialised midwifery programmes were offered at state-funded nursing institutes and collages. Today, thirty-eight educational institutions offer the three-year midwifery diploma programme.

When these new midwifery programmes were introduced, only a few members of the nursing departments responsible for delivering them had any experience of midwifery teaching. There was no provision for the employment of additional, dedicated midwifery teachers. Rather, nursing lecturers were expected to teach on midwifery courses in addition to their existing teaching duties. As such they faced significant challenges in transitioning from teaching nursing to teaching midwifery. To upskill existing midwifery educators, a blended web-based master’s programme for midwifery educators was established. The programme used foreign expertise and an innovative learning environment to build the capacity of midwifery educators in line with government policy [12]. One innovative way to contribute to the capacity building of midwifery educators is through the use of technology. The Internet has made it possible to share information, connect with people on an unprecedented scale [13]. The use of blended learning, which couples web-based learning with face-to-face contact, has been described as contributing positively to students’ achievements in higher education [14–16]. However, there is little evidence to show the distinctive contribution of blended and flexible study programmes to capacity building of midwifery educators in low-resource settings. Therefore, the **aim** of this study was to describe the expectations of midwifery educators in Bangladesh who took part in a blended web-based master’s programme in SRHR and the extent to which these were realized after 12 months of part-time study.

## Methods

### Design

In March 2017 a structured baseline questionnaire was distributed to all participants at the start of the first core course (n = 30) and a second endpoint questionnaire was distributed in May 2018 after they had completed the core courses (n = 29) one year later. At the start of the first core course, five focus group discussions (FGD) were held with the midwifery educators, with a topic guide reflecting the WHO ‘theoretical learning’ domain [8]. Ethical clearance was obtained from the Bangladeshi Directorate General of Nursing and Midwifery on 21 February 2017.

#### Evaluation of higher education for midwifery faculty

Dalarna University is a partner of the United Nations Population Fund (UNFPA) Bangladesh. Since 2016 Dalarna University has supported the government of Bangladesh to increase the capacity of teaching staff at the 38 public colleges in Bangladesh offering midwifery degree programme. Dalarna University staff were responsible for setting up the programme, designing it, teaching on it and monitoring it. They supervised and mentored students using both face-to-face and on-line delivery methods. The current study evaluated the blended web-based master’s programme particularly its topics in sexual and reproductive health and rights (SRHR), its teaching and learning, its leadership and management strategy, its use of recent scientific evidence and the research methodologies.

This programme was not medical, but education/teaching focused. The master’s programme taught the educators about learning styles and other pedagogical approaches that they could use in their teaching. The programme was organised according to Swedish higher educational norms. Here, a one year master’s degree comprise 60 ‘credits’ after batchelor degree comprising 180 ‘credits’. The master’s degrees can be achieved through full-time or part time study. The Bangladeshi one year master’s degree was delivered in part-time mode. Students who completed the five core courses of 7.5 credits each were eligible to receive an ‘Expert Midwifery Educator’ certificate. Those who went on to complete a degree course of 7.5 credits and an independent thesis course (15 credits) were eligible for a master’s degree, awarded by Dalarna University. This programme, alongside the delivery of specialist midwifery training, laced a strong emphasis on critical and reflective thinking and decision-making skills. The curriculum was based on the ICM Global standards for midwifery education (2013) and the WHO Midwifery Educators’ Core Competencies (2014) [7,8].

The programme implemented a blended-learning approach [17,18]. Dalarna University provided web-based teaching that included pre-recorded lectures in English and virtual seminars partly translated into Bangla, as well as electronic library access. Eighty percent of the teaching was delivered via an online learning platform. The remaining 20 percent involved faculty from Dalarna University meeting the midwifery educators/master’s degree students at Dhaka Nursing College, Bangladesh where they ran supervision sessions, held examinations, evaluated the programme and gave verbal introductions to new courses that were delivered in English, although partly translated into Bangla.

### Participants

The students on this programme were all midwifery educators employed by the Bangladeshi government to deliver midwifery programmes to trainee midwives at 15 institutes. They had all trained as nurse-midwives and each one had more than 5 years and less than 30 years of experience in nursing and midwifery. They had taken a one-month course showing them how to implement the midwifery curriculum and how to conduct student assessments. All of them had also taken a six-month intermediate certificate programme in midwifery. Some also held a master’s degree in nursing or public health. Their ages ranged between 45 and 59 and they were able to write in English better than they were able to speak it. All of the midwifery educators (n = 30) agreed to answer both the baseline questionnaire, which examined their expectations at the beginning of the first core course, and the endpoint questionnaire (n = 29), which came after the completion of the core courses and reflected their realizations, their actual experiences of the programme and what they had learned from it. The Focus Group Discussions (FGDs) were part of the baseline and reflected student expectations at the beginning of the first course. The voluntary nature of participation in the study and the confidentiality of any written or verbal contribution was explained before filling in the questionnaires and taking part in the FGD. Oral and written consent was obtained from the participants [19].

### Material and data collection

#### Questionnaire – baseline and endpoint

**Figure 1.**
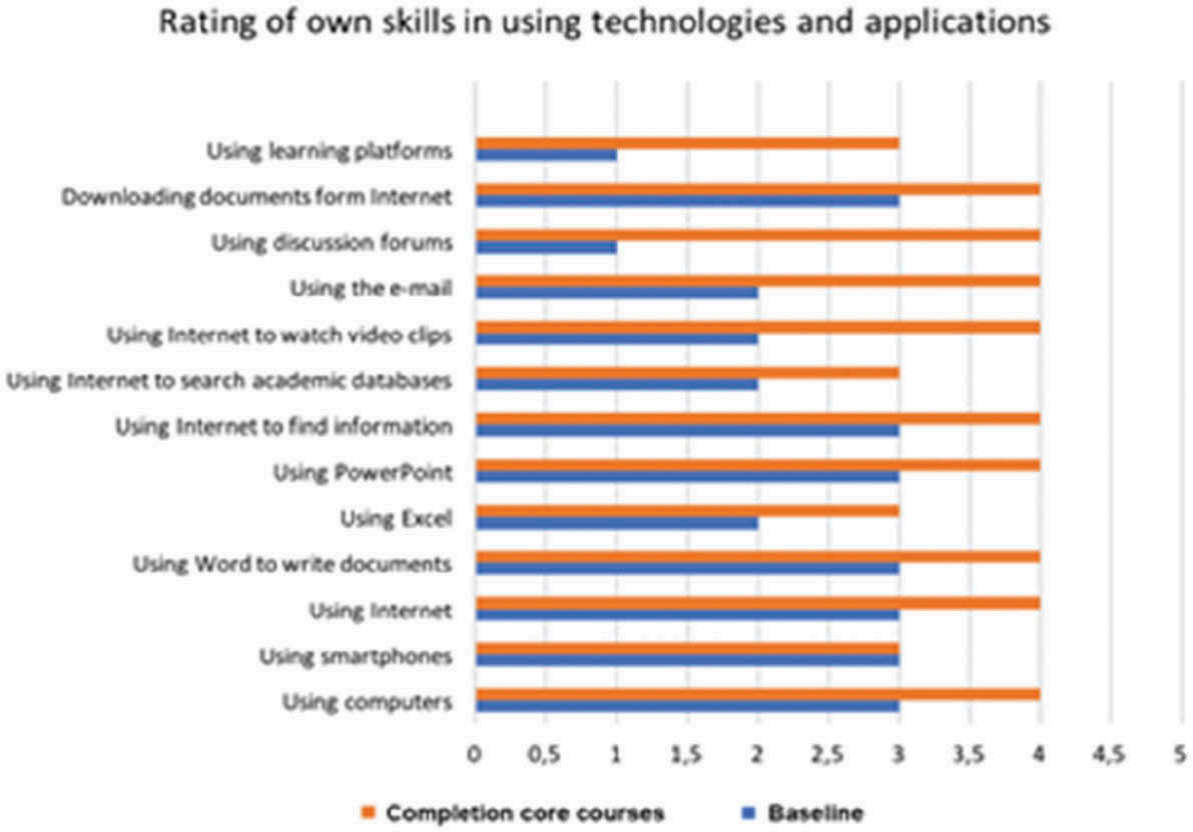
Midwifery educators’ ratings of their own skills in using technologies and applications for selecting effective teaching and learning resources at baseline and completion of core courses. Scale ranging 1 = no skills to 5 = very skilled.

**Figure 2.**
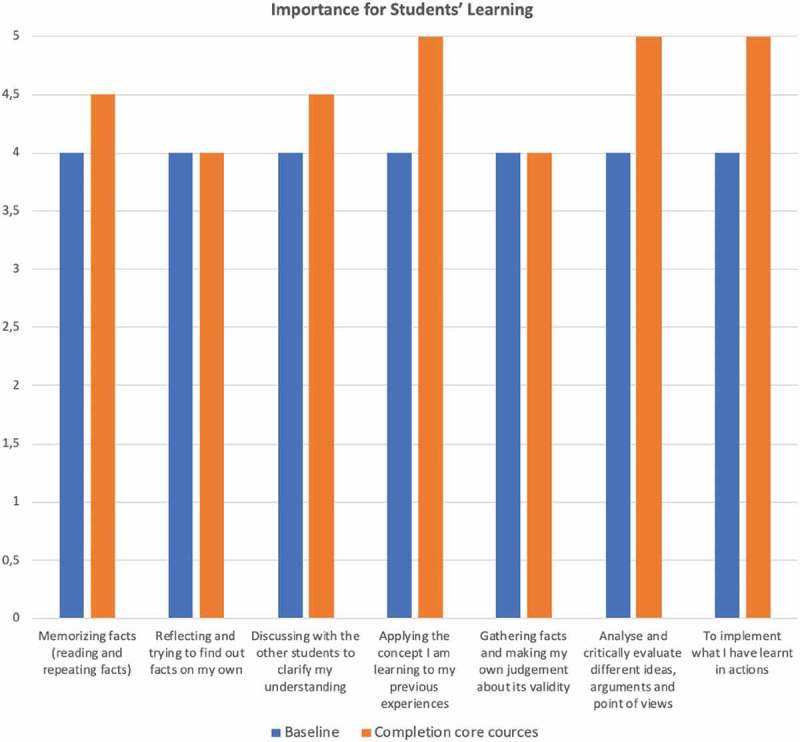
Midwifery educators’ ratings off the importance for students’ learning of different learning styles and approaches at baseline and completion of core courses. Scale ranging from 1 = not important to 5 = very important.

The questionnaire used at both baseline and endpoint consisted of a self-reporting rating scale survey with 10 demographic questions and 24 closed-response alternatives on teaching and learning technologies and didactic teaching in SRHR, using a five-point Likert scale [20]; midwifery educators’ ratings of first, their skills in using technologies and applications for selecting effective teaching and learning resources and, second, the importance of the programme in promoting the effective teaching and learning of their students. The midwifery educators’ skills in using technologies and applicatons were based on a scale ranging from 1 = no skills, to 5 = very skilled (Figure 1). To determine the importanceto the participants of different learning styles for student learning, a scale was used that ranged from 1 = not important to 5 = very important (Figure 2). Two final open questions allowed participants to reflect on, in the baseline questionnaire, their expectations and, in the endpoint questionnaire, what they felt they actually achieved: ‘*Please list the greatest opportunities and benefits that come with the facilitation of a creative theoretical learning environment using technologies and applications’* and, ‘*What greatest hindrances and problems do you see with the facilitation of a creative theoretical learning environment using technologies and applications?*’ In both the baseline and the endpoint questionnaires participants were asked about their access to a computer, smartphone and the Internet with the alternatives yes/no. The questionnaire was administered in a classroom at Dhaka Nursing College and took an hour to complete.

#### Focus group discussions – baseline

Each of the five FGDs contained six midwifery educators and were facilitated by one Dalarna University employee not involved in the programme at the time of the study and UN employees, the latter of whom were not involved in the master’s programme at the time of the study. The topic guide for the FGD was based on ‘Theoretical Learning’, one of the core competencies for midwifery educators outlined by the WHO [8]. *In this the discussions supported the focus of the baseline questionnaire, which focused* teaching and learning technologies and didactic teaching in SRHR. The FGD helped to clarify participants’ expectations and lasted for 50–60 minutes. The ‘Theoretical Learning’ domain includes three competencies: (a) *Incorporate educational strategies to promote active learning*; (b) *Use technologies and applications for the selection of effective teaching and learning resources* and (c) *Recognize and support different learning styles and the unique learning needs of midwifery students*. A principal question for each competency was devised, with follow-up questions such as ‘*Please elaborate on collaboration with other midwifery instructors.’* or *‘What about using e-mails to communicate with the midwifery instructors?’* The midwifery educators in each of the FGD groups were also encouraged to elaborate on what they felt were the greatest opportunities and benefits that emerged from a technology-assisted learning environment and the greatest obstacles and challenges that they saw with this. The FGDs were digitally recorded with the participants’ permission.

## Analysis

Responses to the closed-response alternatives in the questionnaire were analyzed by MH with descriptive statistics, using SPSS version 20. The average of each response alternative was calculated and is presented in Figures 1 and 2. The qualitative data derives from 57 pages of FGD transcripts and 24 pages of responses to the open questions in the questionnaire and was analyzed with content analysis inspired by Elo and Kyngas [21]. The data was read several times. Parts of the text that related to the study’s aim were identified by three Dalarna University employees (KE, MH, MKA), of whom one (KE) had been involved in the master’s programme. Once identified the relevant extracts were then condensed and coded. Based on similarities and differences that were noted between the extracts that related to the study’s key themes, parts of the text were combined and refined. A narrative description of midwifery educators’ expectations and realizations after the completion of five core courses was constructed as the final stage of the analysis process. The process and descriptive outcome of the qualitative analysis was scrutinized by all members of the team. These descriptions and quantitative results were compiled, relating to midwifery educators’ familiarization with technology and how it contributed to their understanding of the learning styles of their midwifery students. .

## Results

### Familiarization with technology

#### Quantitative results

As presented in Table 1, the midwifery educators had lower expectations in terms of their ability to access technology at the beginning of the first course compared to the self-rated outcome after completion of the core courses. Access to a computer and smartphones increased from 26 and 13 at the beginning of the first core course to 29 and 25 after completion of the core courses, respectively. All 29 midwifery educators reported that they had access to the Internet after completion of the core courses, compared to only half of them (15) at the beginning of the first course (Table 1).

**Table 1.** Midwifery educators’ access to a computer, smartphone and the Internet.

	A computer	A smartphone	The Internet
**Beginning of the first course (baseline)**			
Yes	26	13	15
No	1	5	6
**After completion of core courses (endpoint)**			
Yes	29	25	29
No	0	1	0

Figure 1 illustrates the midwifery educators’ ratings of their own ability to use technologies and applications for the selection of effective teaching and learning resources. Their self-rated ability increased in all areas (Figure 1) after completion of the core courses. The most notable increase (from rating 1 to 3) was in regard to using a web-based learning platform, from rating 1 to 4 for discussion forums, from rating 2 to 4 for e-mails and for watching educational video clips from rating 2 to 4.

#### Qualitative results

The participants articulated a number of expectations concerning their familiarity with the use of technologies and the ways in which it could be beneficial to their teaching and professional development. These expectations are presented in Table 2 alongside their realization after completion of the core courses.

**Table 2.** Expectations and realizations related to familiarization with technology.

Familiarization with technology	
Expectations	Realizations
Gain computer knowledge and skills	Being able to download course documents and available resources, such as pre-recorded lectures and handouts for the development of learning materials
Use computers and applications	Communicated with midwifery students using computers and applications
Use the Internet to search for research articles and evidence within the field of midwifery	Used links to video clips and books on the Internet in teaching
Learn English	Improved English language skills

##### Expectations

Most of the midwifery educators expected to acquire new knowledge and skills related to the use of technology. Since the use of computers is an essential part of modern education, students were expected to learn more about computer use during the core courses. The midwifery educators hoped they would gain more computer knowledge and skills at the baseline. Learning how to use computers and applications was the most prevalent expectation midwifery educators expressed when they enrolled on the master’s programme. Midwifery educators stated that they hoped they could learn how to use the Internet to search for research articles and the latest evidence-based practice and to learn about the field of midwifery. After this they hoped they would learn how to teach this updated knowledge to their midwifery students and be able to do so using improved English language skills. A participant in FGD 1 said:

*“Number one is IT knowledge and skills and the second is knowledge about the subject sexual and reproductive health and rights (SRHR). Moreover, we will learn research and evidence-based care and gain English language skills”* (FGD1).

Some midwifery educators, however, expressed difficulties about incorporating technology and applications into their regular teaching practice because of a lack of access to the Internet in the office as well as a heavy workload which made the time necessary to learn a new skill overwhelming. Another participant in FGD 1 stated:

*“During office hours, it is difficult to access the Internet, but we could do it in the evening from home. We have workloads and we have to do everything at work: for example, preparing for classes and student exams, mentoring the clinical practice sessions and many other things. Despite the obstacles, we hope that we will do well”* (FGD 1).

Some midwifery educators were, from the beginning of the first course, already well experienced with technology and applications. They used the Internet to search for information and were active on social media platforms. According to one participant,

*“I am skilled in Word, Excel, PowerPoint and web-browsing. I use the computer to prepare my lecture, and I use my mobile phone to post on Facebook and for video calls by Skype for communication with my family, students and colleagues”* (FGD 4).

#### Realizations

Being able to download course documents and resources, such as pre-recorded lectures and handouts, was considered an important achievement in terms of the midwifery educators’ ability to develop learning materials that in turn enhanced their midwifery students’ learning.

“What the midwifery students study in the theoretical courses and experience in clinical practice and in skills lab I now manage to combine when I develop learning material, for instance to guide a group’s work” (Questionnaire 14).

English was the language used in face-to-face classes, in web-based seminars and in discussion forums. Midwifery educators felt this experience of using English enabled them to improve their English language skills:

*“The activities in the discussion forum with national and international colleagues increased my English vocabulary and this, in turn, enables me to provide students with information from all around the world about midwifery”* (Questionnaire 28).

The midwifery educators learned how to use links to video clips and e-books on the Internet in their teaching. Some preferred to use handouts, write on the blackboard and other more “

traditional ways of educating students. However, even these midwifery educators learned how to communicate with their midwifery students in less traditional ways. A positive aspect that was brought up was that the web-based and part-time nature of the programme enabled students to work and study at the same time. One of the participants stated:

*“You are not separated from your family if you do not live in Dhaka as would be the case with campus-based education in Dhaka and this provides the opportunity to learn and work at the same time”* (Questionnaire 3).

However, this meant that the midwifery educators needed to take active responsibility for their studies.

### Contribution to midwifery students’ learning

#### Quantitative results

As presented in Figure 2, the midwifery educators were asked to rate what they felt was the importance of different learning styles and to demonstrate the extent to which they recognised the unique learning needs of their students in the baseline as well as in the endpoint questionnaire. The importance of each aspect was rated with a median value of 4 in the baseline survey; all aspects were considered equally important (Figure 2). The midwifery educators found that their knowledge of learning styles helped them to diversify their approach to teachingbenefitting their students.

#### Qualitative results

The participants expected that their involvement in the programme would contribute to the learning of their midwifery students in different ways. These expectations are presented in Table 3 together with the realizations upon completion of the core courses.

**Table 3.** Expectations and realizations related to contribution to midwifery students’ learning.

Contribution to midwifery students’ learning	
Expectations	Realizations
Enabled to support student’s learning	Increased capacity to use different learning content and pedagogical approaches
	Inspired to use new pedagogical approaches
	Used learning outcomes in the course plan to determine the assessment criteria for midwifery student assessments.
	Promoted the active learning/creative learning strategies of midwifery students
	Used computerized assessments

##### Expectations

The midwifery educators were not accustomed to implementing a range of educational strategies in their teaching with the goal of promoting students’ learning. One of the main expectations the educators had when starting the programme was learning how to implement strategies for teaching in terms of technology, methodology and pedagogy that facilitated the development of long-term quality midwifery care in Bangladesh.

*“We will learn lots of technology, methodology and pedagogy including online presentations, which is good, a mixture that we can later use in the classroom. We will also learn to communicate, which will be innovative and benefit our students’ learning”* (FGD 2).

They expected they would be taught how to do online searches so that they could find new evidence-based research in their field of knowledge. They would be shown new web-based teaching methods that would enhance their own practice. They hoped to be aboe to use this knowledge so that they could ensure their midwifery students upon graduation, could ensure local midwives provided better evidence-based care to women and newborns as a result This was expressed in the baseline questionnaire:

*“I will share ideas and exchange information using different web-based activities, social media and telecommunication. Finally, I would like to say I want to improve professionalism and contribute to midwifery students’ learning”* (Questionnaire 16).

##### Realizations

By the end of the study the midwifery educators felt they had increased their capacity to use different learning content and pedagogical approaches. They were inspired by the Swedish midwifery educators to try new pedagogical approaches. Many were inspired by the way their Swedish teachers used assessment criteria and linked these to the learning outcomes in the course plan. Because they were working at the same time as studying, the midwifery educators were able to use these skills to assess their own midwifery students against assessment criteria. The midwifery educators were able to use their new knowledge of computerized assessments, for instance, to promote their students’ active learning on a daily basis. As one participant noted,

*“We gained knowledge about assessment criteria as a “leading star” throughout each course. With this knowledge, we can teach our students properly so that they can develop their competency. Previously in education, we didn’t use assessment criteria or computer labs, and now we are doing questionnaires using the computer, and we are doing an assessment and monitoring our midwifery students through the computer, so through this course hopefully we have developed a lot more pedagogical skills”* (Questionnaire 4).

The courses were a resource that increased students’ knowledge of midwifery and how to teach it. the midwifery educators became more confident teachers, comfortable communicating the central aspects of midwifery practice to their students using both traditional as well as new and innovative methods. The midwifery educators saw the master’s programme as an opportunity to participate in the international midwifery movement by way of the Internet and felt it would inspire them when they educated their own midwifery students. One midwifery educator wrote in the endpoint questionnaire:

*“I can communicate with others from other countries from time to time, to establish partnerships with others in other countries and get inspired to implement the SDG to the best of my capacity when I teach the students”* (Questionnaire 11).

### Tools to empower midwifery students

In this section the qualitative data relating to midwifery educators’ expectations in the third and final area is explored. Midwifery educators expressed a range of expectations that related to their ability to empower their students’ theoretical and clinical learning. These are presented in Table 4, along with their thoughts about the extent to which this had been realised after completion of the core courses.

**Table 4.** Expectations and realizations related to tools empowering midwifery students.

Tools to empower midwifery students	
Expectations	Realizations
Make midwifery students aware of the situation for women and learn about?? tools to empower them as young women	Being able to lead ‘Awareness Sessions’ for midwifery students using critical thinking, reflection and decision-making skills
Knowledge and tools on how to educate midwifery students about women’s rights.	Realized their own potential to empower others

#### Expectations

The situation for girls in Bangladesh has gradually changed as a result of an increasing number of women entering higher education. However, the midwifery educators strongly felt that, as young women, midwifery students needed to be empowered if they were to be able to function as working professionals who dealt with women on a daily basis. The midwifery educators, therefore, expected the core courses to provide them with tools that they could use to educate their midwifery students in women’s rights. *“Midwifery students will benefit from the subject Sexual and Reproductive Health and Women Rights* (Questionnaire 16). The midwifery educators described the situation for women and girls in Bangladesh in the FGDs. For example:

*“Our midwifery students are girls, and they will learn how to reflect and think and decide, and that is not appreciated within society. We need to make them aware of their rights so that they can counsel mothers-in-law so that their behavior towards their daughters-in-law changes, for instance”* (FGD 2).

Midwifery educators felt that their students needed to be made aware of the women’s rights and of the proper care that should be provided to every woman and child during pregnancy and childbirth in Bangladesh and worldwide in a variety of ways.

*“I am confident that if we get trained in how we train our midwifery students properly about the care that should be provided to every woman and girl, then they will serve the pregnant mothers in antenatal care and ensure exclusive breastfeeding. I am taking good care of my students and I teach them in a variety of ways how to take care of women in the antenatal ward and how to ensure exclusive breastfeeding”* (FGD 2).

#### Realizations

The midwifery educators stated how exercises in critical thinking, reflection and decision making had transformed their professional outlook. Whereas before they had held more conservative views of reproductive health, now they were more open to women’s rights and were willing to challenge their students to develop a similar respectful attitude towards women and newborns. *‘Being made aware sessions’*, as one of the participants called them(Questionnaire 30 endpoint), took place across the programme, through in-class discussions, reflections on film clips or news reports from the internet. Together they raised midwifery educators’ awareness of the state of midwifery care, including the ongoing presence of social stigma and discrimination. As described in one questionnaire: *Critical thinking, reflection and decision-making are particularly important for girls, and the midwifery students are girls, and will help to reduce the social stigma and discrimination towards women* (Questionnaire 29).

By the end of the study period the midwifery educators realized the potential they had to empower their students in their clinical practice. The master’s courses enabled the midwifery educators to recognize the importance of communication [do you mean discussion and debate?] and time spent with students as a way of guiding and empowering them. Together with their students, they could help to close the gap between theory and practice and to provide respectful care. *‘I have realized the midwifery students need support in clinical practice, they need to be guided to actually provide proper care* (Questionnaire 10)’.

As part of their desire to empower their students, midwifery educators also recognised the importance of technology and applications to enhance student learning. They recognised that money, language skills, family support and information skills were essential foundations for developing young women’s sense of their human rights. However, they also recognised that students needed to be able to use a computer and to access the internet if they were going to take full advantage of educational opportunities and reach their full potential. As one midwifery educator wrote:

*“You need enough time, money, your own computer and the necessary expertise in technology and English skills to benefit from the technology. Still, a lack of electricity, weak Internet connectivity, and language barriers are hampering women’s education”* (Questionnaire 24).

Thus, midwifery educators in this study expressed the need for better internet access at their colleges. An improved learning environment for midwifery students was, for them, dependant on improved library and internet services.

## Discussion

The use of a blended learning approach, when web-based learning is coupled with face-to-face contact, has previously been described as contributing positively to students’ achievements in higher education [14–16]. When the midwifery educators in this study rated their own skills in using technology and applications, they rated improvement in all areas during the time they took the core courses. They notably increased their use of the online learning platform, discussion forums, e-mails and educational video clips. They used their growing knowledge of the online learning environment to create online communities and networks that supported their own learning and gave them ideas to use with their own students. This is in line with Omer et al. [13], who described experiences in a university in Somaliland [13]. Further inquiries and analysis of the qualitative data revealed that some of the midwifery educators were already skilled in using mobile phones and computers, both for work and entertainment, whilst for others their use presented a significant challenge. This resistance to technology might derive from wider attitudes towards change within the midwifery educator profession, and should be investigated further so that there is a better understanding of how to teach these students. Some participants mentioned that the use of technology was stressful whereas others adapted easily to the new requirements. Nevertheless, the midwifery educators’ ability to use technology generally improved during the core courses. Access to smartphones increased and all midwifery educators reported that they had access to the Internet after one year of education, and their use of the internet went up. This had to do with their motivation to improve their own teaching in the workplace. With determination, they all managed to use the Internet for work purposes. Our results were consistent with Fullerton et al. (2013) and Bharj et al. (2016) [4,22], who found that many midwifery educators are not prepared for the role because they lack the opportunity to develop their professional competencies and skills. Being able to use technology is important for a midwifery educator. If a comprehensive programme for capacity building is implemented, such as is the case in Bangladesh and Somaliland, midwifery educators will improve their competence, not only in the core subjects around sexual health and midwifery practice but also in those that concentrate on teaching and learning pedagogies and the use of technology [17,18].

The development and implementation of a comprehensive programme to build the capacity of midwifery educators in Bangladesh has enabled midwifery educators to take responsibility for their own teaching and learning and to address the different learning styles of their midwifery students. Participants in this study stated that midwifery educators used their experience to inform their students and made them want to inculcate a more respectful attitude towards women and newborns. This is consistent with the evaluation of the master’s programme developed and implemented in Somaliland [18]. The web-based instruction provided by the capacity-building team from Sweden created a learning environment where student-centred learning was the foundation. Co-creation involving international partners with local universities, midwifery associations and NGOs employing different ways to build the capacity of midwifery educators has been described in Bogren’s article [12]. Our study is unique in that it evaluates the use of blended web-based learning as a tool to reach midwifery educators in both urban and rural low-resource areas. It has provided these educators with a flexible educational model that allows them to continue working while developing their professional practice.

Furthermore, the fact that it was women who had opportunity to study online is expected to have a positive impact on gender equality and on efforts to achieve equity in education [17]. The web-based learning environment fostered reflective and critical thinking among the midwifery educators in this study, which it is expected they will go on to foster in their students It is anticipated that the evidence-based midwifery teaching and the simulation based training the educators received will enable them to develop critical thinking skills among their students.

Our results are concurrent with research stating that competent midwifery educators who contribute to the learning of midwifery students can translate the vision of universal provision of quality midwifery care into reality [1,23]. The results are also in line with the call by the WHO for a strong and effective midwifery workforce that is able to respond to the twenty-first century priority of providing women and newborns with quality maternity and newborn care [24]. There is a global call for increased resources to be set aside specifically for low-resource settings where the need to identify interventions to enhance the quality of education and the development of the learning environment in general is crucial [25].

## Strengths and limitations

The description of the core competencies of the midwifery educator, as stated by the WHO [24], served as a useful base for the development of the self-rated questionnaires and topic guide for the FGDs [24]. The main limitations of and threats to the credibility of this study were the self-rated questionnaire, the FGD and the language barrier. Courses in English and language control prior to enrolment in similar programmes is therefore recommended. Despite these obstacles, the quantitative and qualitative results complemented each other, thus strengthening the trustworthiness and transferability of the study’s results. Furthermore, the researcher (MH), an expert on web-based learning, and a professor in SRHR (MKA) took part in the steps of the research process and challenged the rest of the team involved with the master’s programme to be aware of their preconceived notions and the response bias. As such, they acted as outsiders in the data collection, analyses and interpretation of the results. With this in mind, despite the potential response bias [20], the study results can be transferred with caution to other settings. The results can be useful in the planning, implementation and evaluation of similar initiatives related to the capacity building of midwifery educators.

## Conclusions

Building the capacity of midwifery educators in Bangladesh has according to the midwifery educators enabled them to take responsibility for their own teaching and learning. Midwifery educators have taken part in a creative learning environment which has developed their engagement in teaching and learning. They have done this using a blended learning model which combines online learning with face-to-face contact. This model can be scaled up in low resource and remote settings. As an educational framework it can be easily adapted so that the WHO midwifery core competences could guide the capacity-buiding initiatives of other midwifery education programmes.

## Paper context

We describe and evaluate a comprehensive model for the building of midwifery educator capacity in Bangladesh using a blended learning programme. This educational model has contributed to midwifery educators’ skills and increased their ability to take responsibility for their own teaching and learning. It is expected these skills will, in turn, enhance the learning of future midwifery students. Using web-based and face-to-face learning to build the capacity of midwifery educators may be scaled up and implemented in low-resource settings and other less accessible places.
